# Symptomatic Carotid Artery Thrombosis in a Patient Recently Recovered From a COVID-19 Infection

**DOI:** 10.7759/cureus.18626

**Published:** 2021-10-09

**Authors:** Ilkin Bakirli, Jan Tomka, Marian Pis, Hasan Bakirli, Gultakin Bakirova, Matej Osusky, Andrej Gazi, Ifrat Bakirov

**Affiliations:** 1 Vascular Surgery, National Institute of Cardiovascular Diseases, Bratislava, SVK; 2 Vascular Surgery, Slovak Medical University, Bratislava, SVK; 3 General and Oncology Surgery, Cyril and Methodius University Hospital, Bratislava, SVK; 4 General and Oncology Surgery, Comenius University, Bratislava, SVK; 5 Critical Care, King Saud Medical City, Riyadh, SAU; 6 General Surgery, Al Imam Abdulrahman Alfaisal Hospital, Riyadh, SAU

**Keywords:** cytokine storm, anticoagulation, arteriotomy, angiography, carotid thrombectomy, ischemic stroke, covid-19 infection, thrombosis, carotid artery

## Abstract

Coronavirus disease 2019 (COVID-19), caused by severe acute respiratory syndrome-coronavirus 2 (SARS-CoV-2), was initially discovered in December 2019 in China and rapidly spread all over the world to become a pandemic. The most common symptoms of a disease are fever, cough, generalized body ache, weakness, dyspnoea, nausea, vomiting, and diarrhea. Among vascular complications of COVID-19, the venous thrombotic complications, like pulmonary embolism and lower limb deep veins thrombosis, are not uncommon. But data about arterial thrombotic complications of COVID-19, especially carotid thrombosis, are still limited. We are describing a case of stroke due to thrombosis of the right carotid arteries, in a patient who had recovered from asymptomatic COVID-19.

A 66-year-old male with arterial hypertension presented to the emergency department with a history of repeated collapse, dysarthria, weakness in the left extremities, and a drop in the left angle of his mouth (National Institutes of Health Stroke Scale [NIHSS]-4). The patient was swabbed for COVID-19 which was negative. A computed tomography angiography (CTA) was obtained which showed thrombosis in the branching point of the brachiocephalic trunk (BCT) continuing into the right subclavian artery (SA) and also into the right common carotid artery (CCA), with a subtotal occlusion of the right CCA, extending into the internal carotid artery (ICA) as well. From the apical lung tissue caught during the CT scan, bilateral, irregular widespread ground-glass opacifications, as well as consolidations and small reticular changes were seen in the lungs, which is typical for COVID-19 infection. A quantitative antibody test for COVID-19 infection was performed with the results showing a strong positivity for IgG antibodies, indicating previous COVID-19 infection. The patient was indicated for a standard carotid thrombectomy, which was performed without complications.

It seems that one of the important factors that led to the formation of the thrombus in the carotid arteries was COVID-19 infection-induced inflammation in the atherosclerotic carotid vessels and generalized hypercoagulability as well as hyperviscosity.

COVID-19 infection is an independent and important risk factor for the formation of an arterial thrombus during the acute illness and in the early post-COVID-19 period also, regardless of the severity of its course. Prophylactic anticoagulation is needed not only at the time of acute illness but also at the early post-COVID-19 time.

## Introduction

Coronavirus disease 2019 (COVID-19), caused by severe acute respiratory syndrome-coronavirus 2 (SARS-CoV-2), was first identified in December 2019 and has since become a worldwide pandemic [1]. Although primarily it is a respiratory disease, with symptoms such as fever, cough, and dyspnoea, venous and arterial thrombotic complications have also been reported. Four cases were described with a severe form of thrombotic event with aortic involvement [2]. The exact relationship between the occurrence of arterial thrombotic events and SARS-CoV-2 remains unclear [3]. However, COVID-19 is associated with causing a hypercoagulable state. COVID-19 is also associated with a severe inflammatory response, which affects atherosclerotic plaque vulnerability and promotes a thrombogenic environment [4-9]. Patients who develop a severe disease have been found to have markedly increased levels of inflammatory cytokines, especially interleukin-6 (IL-6), what is termed a “cytokine storm” [10]. Neutrophil activation is another important feature of COVID-19, as activated neutrophils are the first to respond to the invasion of pathogens [11]. Neutrophils that fail to extravasculate are partially broken down in the circulation. These floating neutrophil cells, also known as low-density granulocytes, are prone to release their contents, with antimicrobial agents stored in their granules, a process known as neutrophil extracellular trap formation (NET). Excessive NET formation leads to aggregate formation and this causes occlusion of vessels, with prothrombotic coagulopathy involved as a mechanism [12,13]. Interestingly, the patient described in this case study had an asymptomatic course of COVID-19, therefore possibly not exhibiting a cytokine storm.

A particular subtype of acute ischemic stroke, large-vessel occlusion (LVO) is characterized by occlusion of a major extracranial or intracranial vessel and it represents 24-38% of all acute ischemic strokes [14]. One of the most important causes of LVO is an artery-to-artery embolism, usually due to the presence of an atherosclerotic plaque or thrombosis. Vulnerable plaques are the biggest concern in this matter as they have a higher chance of undergoing rupture and causing an embolism. It is inflammation, which COVID-19 also stimulates, that plays a big role in determining the vulnerability of the plaque by multiple cellular and molecular mechanisms [15,16].

## Case presentation

This case is of a 66-year-old male, former smoker, with a history of stage 1 European Society of Hypertension (ESH)-European Society of Cardiology (ESC) arterial hypertension and bronchial asthma, who presented to the emergency department with symptomatology indicating a stroke. At home, the patient recalled collapsing twice in the span of 45 minutes. After the second collapse, the patient complained of worsened mobility with weakness in his left upper and lower extremities, the angle of his mouth dropped on the left side and he complained of dysarthria as well (National Institutes of Health Stroke Scale [NIHSS]-4). The patient’s relatives claimed that he fell onto his back without injuring his head.

**Figure 1:**
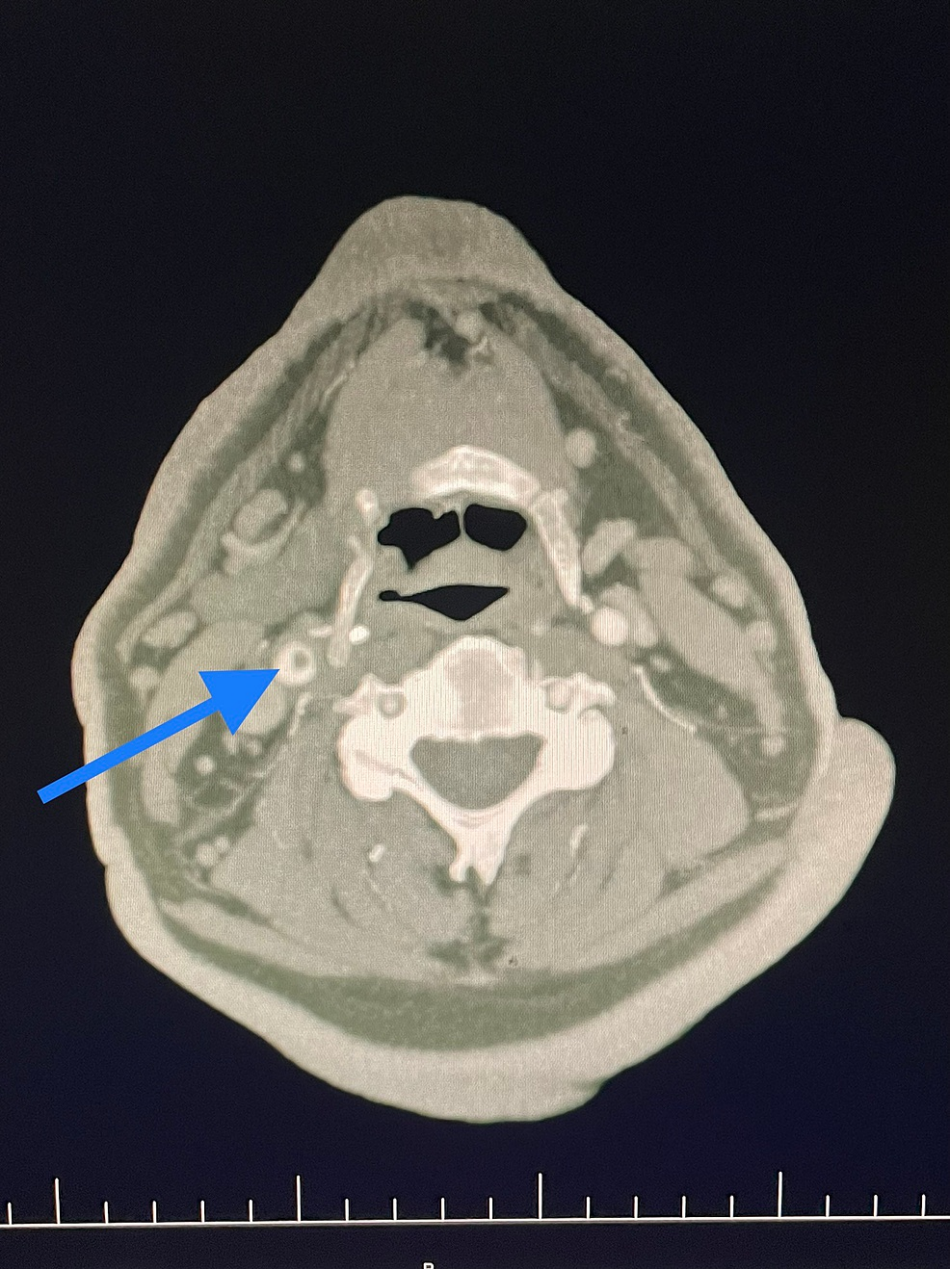
Axial view computed tomography angiogram (CTA) image of the carotid arteries showing a thrombus in the right common carotid artery extending into carotid bifurcation.

**Figure 2:**
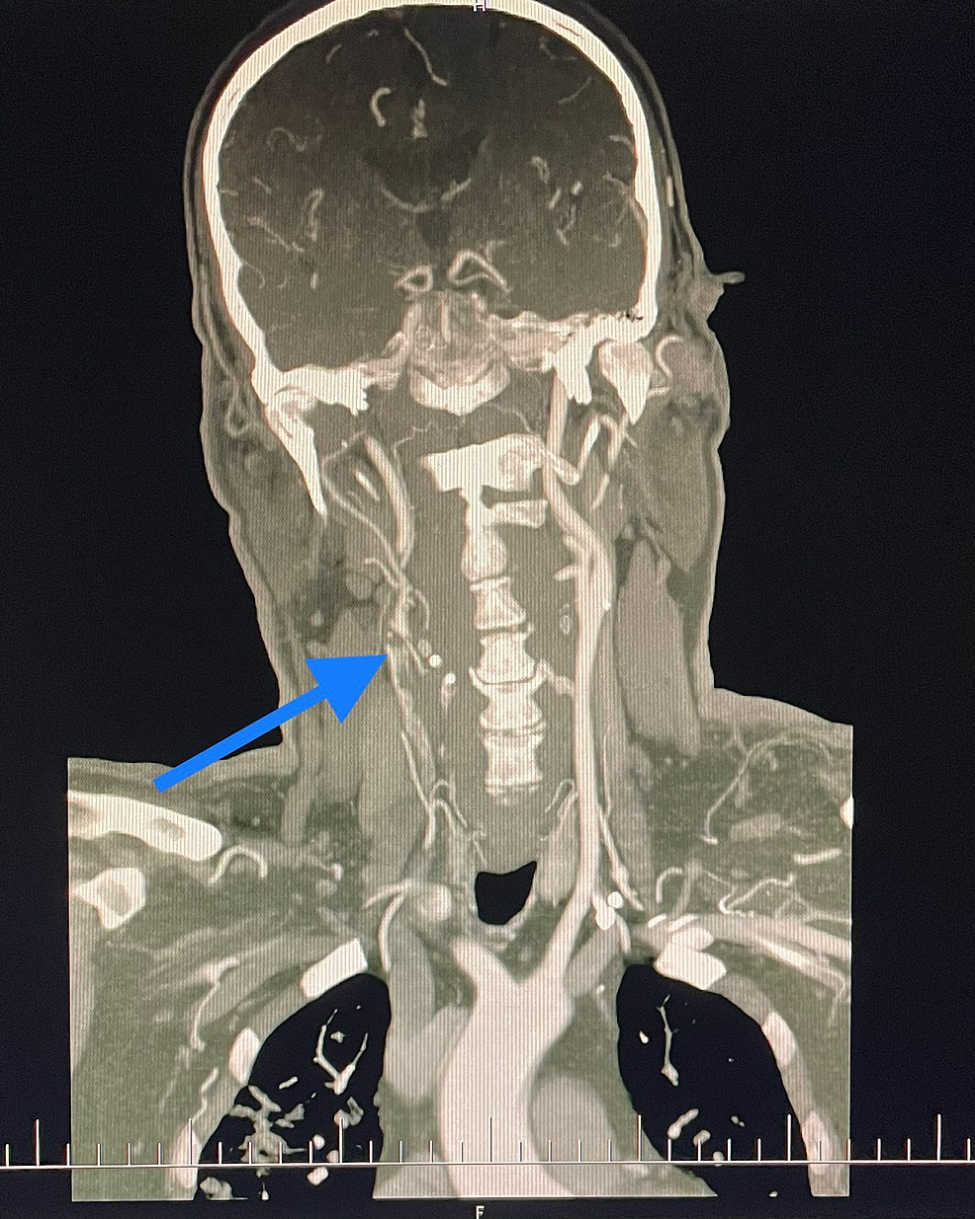
Coronal view computed tomography angiogram (CTA) image shows thrombus in right common carotid artery extending into carotid bifurcation

Upon arriving at the hospital, the patient was stable, conscious, and oriented in time and space. Initial blood analysis was done and every parameter, including coagulation profile, was within the normal range. The patient was then swabbed for COVID-19 antigen and reverse transcription-polymerase chain reaction (RT-PCR) tests as per Slovakia’s guidelines for every patient being admitted to the hospital. The antigen test result was interpreted at 15 minutes as negative. The RT-PCR result came back as negative as well two days later. The patient had no respiratory symptoms and had no relevant history of exposure to a COVID-19-infected individual. The patient was not vaccinated against COVID-19. An urgent computed tomography angiogram (CTA) was obtained in the emergency department. In the native phase of the CT, a pathological hyperdense lesion measuring 12mm was noted in the pons. Supratentorial, in the area of the falx cerebrii, small subarachnoid hemorrhages (SAH) were seen in two points, measuring up to 3mm each, without evidence of bleeding into the infratentorial area. A thrombus was noted in the branching point of the brachiocephalic trunk (BCT) continuing into the right subclavian artery (SA) and also into the right common carotid artery (CCA), with a subtotal occlusion of the right CCA, extending into the internal carotid artery (ICA) as well (Figure 1, 2). Partial occlusion of the distal basilar artery due to a thrombus was also seen. The imaging findings were consistent with thrombotic stroke, likely owing to a COVID-19 related hypercoagulable state in an ex-smoker patient, with mild hypertension.

As an additional finding, from the apical lung tissue caught during the CT scan, bilateral, irregular widespread ground-glass opacifications, as well as consolidations and small reticular changes were seen in the lungs, which is typical for infection with SARS CoV-2. On the basis of this finding, a quantitative antibody test for COVID-19 was performed. The results after two days showed low levels of IgM antibodies but a strong positivity for IgG antibodies, indicating COVID-19 infection in the recent past.

The patient was managed by the neurology team and put on aspirin, Cerebrolysin, and atorvastatin. The risks of adding low molecular weight heparin (LMWH) to the treatment outweighed its benefits, so it was excluded.

In accordance with the CT findings, interventional radiologists were consulted for an interventional thrombectomy of the basilar and left vertebral artery. However, an interventional thrombectomy was contraindicated due to the mildness of neurological deficit and the due to high risk of embolization. Simultaneously, the neurosurgical department was consulted due to the SAH and they did not indicate any invasive procedure as well due to the mildness of the bleeding but recommended a magnetic resonance angiography (MRA) scan of the brain. The next day MRA of the brain was performed, which demonstrated similar findings to the CT scan.

A USG examination of the carotid arteries was also performed, with the findings concluding an unstable ulcerated plaque in the right CCA.

Ten days after admission, the patient was transferred from the neurology department to the vascular surgery department in the National Institute of Cardiovascular diseases in Bratislava, where the patient was indicated for a carotid thrombectomy due to the risk of a repeat of stroke or thromboembolism.

**Figure 3:**
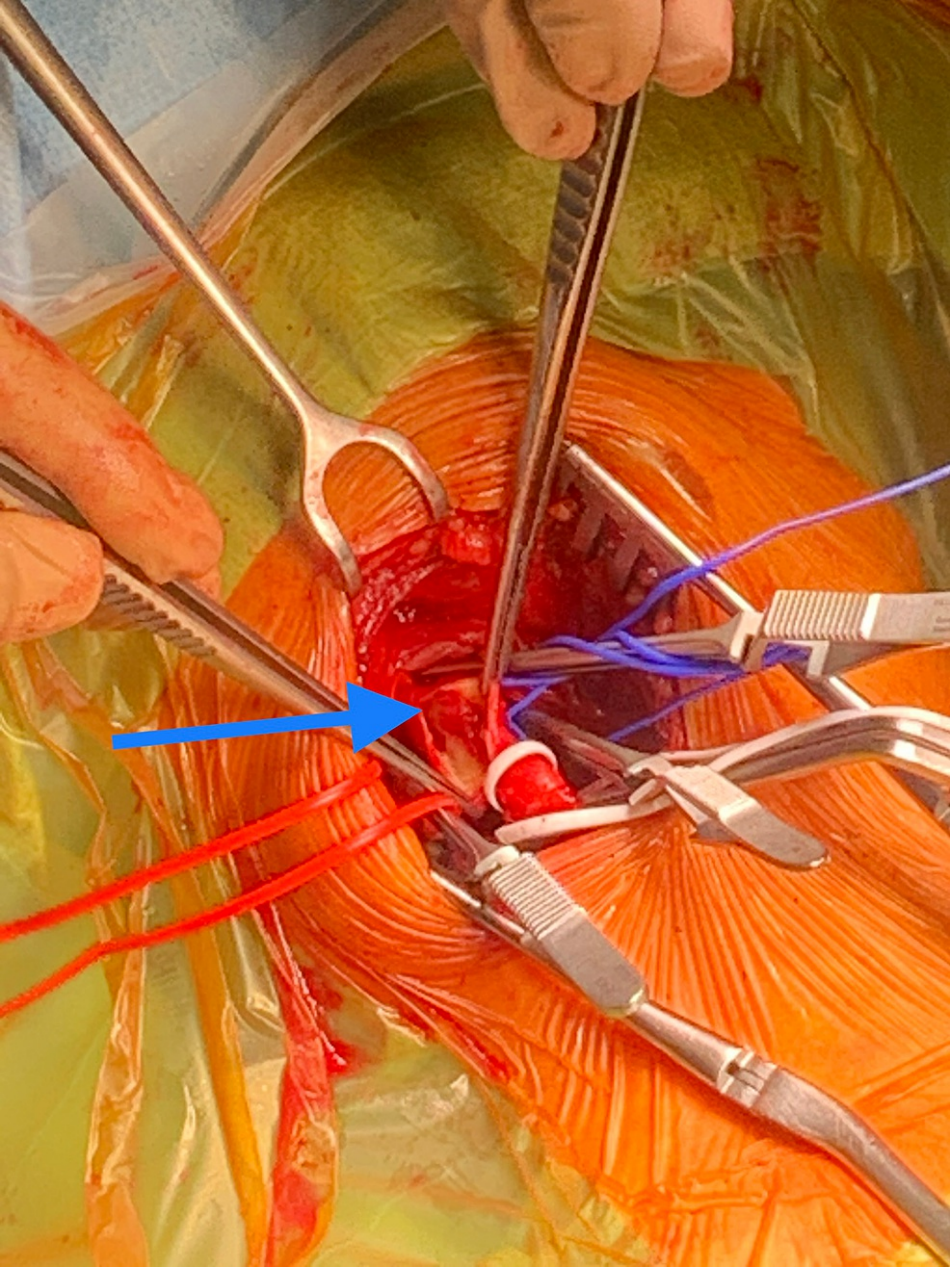
Intraoperative finding: thrombus in the right common carotid artery extending into the internal carotid artery

In the operating room, the patient was draped and scrubbed from the sternal notch to the earlobe. Under the cervical block, a standard vertical incision was made in the anterior aspect of the sternocleidomastoid muscle. After dissection in layers, the carotid bifurcation was located and then controlled with vascular loops both proximally and distally. A lower than the usual dose of systemic heparin (5000IU for a 90kg patient) was administered as a precautionary action to avoid re-bleeding into the intracranium. After three minutes of heparin circulation, the carotid arteries were clamped, the ICA first to avoid embolization, and a longitudinal arteriotomy was made on the CCA extending to the ICA, which revealed an occlusive thrombus (Figure 3). 

The thrombus was easily and in whole removed with DeBakey forceps. Thrombectomy with Fogarty catheters F2 and F3 distally and F6 proximally was performed, removing another small thrombus from the proximal part of the CCA. Blood inflow and backflow were checked by releasing the clamps, which were both optimal. The arteriotomy was closed by a primary suture without narrowing the lumen. Perfect hemostasis was achieved by applying Floseal hemostatic foam to the wound, Redon drain was inserted and the wound was closed in layers.

**Figure 4:**
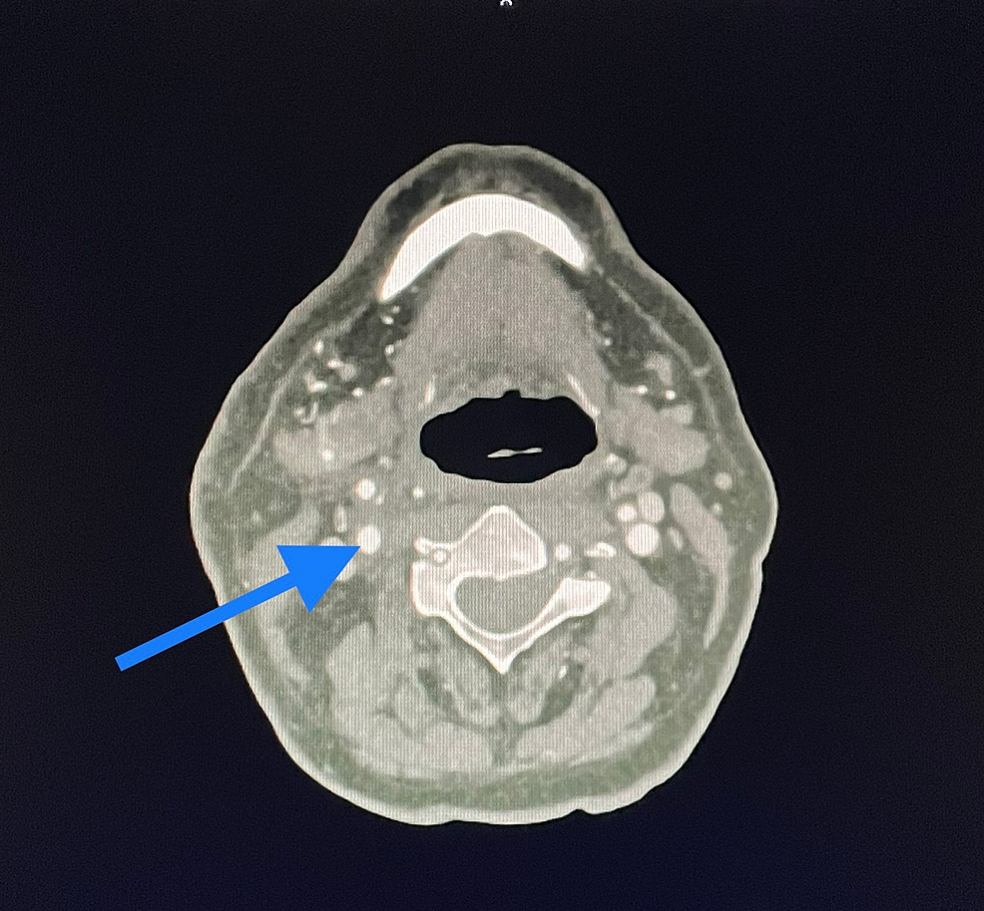
Control CT image shows no residual thrombus in right carotid arteries.

The operation ran without complications, the patient did not manifest any new neurological symptoms and was transferred to the postoperative ward. Redon drain was removed after 24 hours. Fraxiparine 0.6 ml twice daily was added to the treatment. A control CT scan was performed on the second postoperative day that concluded no residual thrombi in the CCA, nor in the aortic arch (Figure 4).

The postoperative period was uneventful. The patient was discharged to home on the fifth postoperative day with aspirin 100 mg/24h, (as well as antihypertensive therapy). The early postoperative review showed wound healing by primary intention, and the next review as an outpatient after two months revealed improvement in the neurological state of the patient, no neuro-deficit was found.

## Discussion

Thrombotic and thromboembolic complications are now well known to be associated with COVID-19, however, mostly occurring in the venous system and in the lower limbs. In a systematic review of 27 studies involving 90 patients conducted by Cheruiyot et al., lower limb vessels thrombosis accounted for 39% of all thrombotic events in critically ill COVID-19 patients [4]. Most likely one of the misbalancing factors that led to the formation of the thrombus in the carotid arteries, and hence an ischemic stroke, was COVID-19 induced prothrombotic inflammation in the atherosclerotic carotid artery and generalized hypercoagulability and hyperviscosity. To date, a few studies reported carotid artery thrombosis and stroke as complications of COVID-19 [17-19]. In the largest series with six carotid thrombosis, atherosclerotic plaque rupture along with the pro-inflammatory state caused by COVID-19 were thought to be the cause of thrombosis, which correlates with the findings in our study [9]. In the same study, several patients suffered only from a mild respiratory illness, suggesting that the inflammatory and coagulopathic complications of COVID-19 might not correlate with the severity of the respiratory symptoms, which also corresponds to our patient. Nannoni et al. performed a systematic review and meta-analysis to investigate the relationship between COVID-19 and stroke [20]. The median age was 65.3 (61.4-67.6) years, and the majority was male (62.4%). Vascular risk factors were common: hypertension (62.2%), diabetes mellitus (36.7%), and dyslipidemia (25.2%). 

## Conclusions

The following important points are notable from this study. Firstly, the patient had an asymptomatic course of COVID-19 infection and at no point was his condition critical, which excludes the theory that only severe cases of COVID-19 are at risk of thrombosis. Secondly, prior to this event, the patient was never treated for any venous or arterial thrombosis, which proves that COVID-19 infection itself is an important risk factor for the formation of a thrombus in the period of acute illness and early post-COVID-19 time. However, it is likely that due to the underlying atherosclerotic plaque the patient was more susceptible to thrombotic occlusion of the carotid artery. Prophylactic anticoagulation is indicated in the acute COVID-19 illness period as well as at the early post-COVID-19 time, especially in aged patients with preexisting cardiovascular disease, like hypertension, diabetes mellitus, dyslipidemia, and ischemic heart disease.
